# Uterine Polypus

**Published:** 1855-05

**Authors:** George Higgins

**Affiliations:** Aurora, Illinois


### ART. III.—*Uterine Polypus.* By George Higgins, M. D., of Aurora, Illinois.

I was called January last to Mrs C. A., seventeen years of age, had been married seven months; was about four months advanced in pregnancy. Two days before I was called, she rode into the country, a distance of five miles over very rough roads in a lumber-wagon. Before arriving at the place of destination, she began to suffer pain in the lower portion of the abdomen and lumbar region, requesting the driver to stop in order that she might get out of the wagon, but he being intoxicated at the time, refused to do so, and she was compelled to remain in that uncomfortable position. She was taken from the wagon, but uterine pains had already commenced with profuse hœmorrhage. After twelve hours she aborted. Hœmorrhage continued up to the time I was called, which was two days after the ride. I found the patient cold, colorless, nearly pulseless frequent fainting, laborious breathing, and flowing steady and profuse. I made use of the usual means to arrest the hœmorrhage, such as external cold over the region of the uterus and commencement of the vagina, and other means as far as time would permit. But to no purpose.—Then I proposed making a vaginal examination. It was objected to. I told the friends the importance of doing it, as the case would prove fatal in a very short time if left to itself, when consent was given. I soon discovered protruding from the uterus, a tumor in appearance soft to the finger, and further examination detected its attachment to the side of the uterus by a nick or pedicle of less diameter than the polypus. At this time, hœmorrhage was profuse. I saw that a speedy removal of the tumor was necessary, as a few minutes more would terminate the patients existence. Then it was as to the best mode of doing it. As there was no time to be lost, I seized it between the fingers, and by a manipulating, rotating, twisting, and pulling operation, I succeeded in bringing it away. The flowing immediately stopped.

The patient recovered as rapidly as is usual in ordinary cases of abortion with the same loss of blood.

On examination of the polypus, I found it to be about eight inches long, by two in diameter, and attached as before stated.

I found the polypus to be hollow, and in the cavity which held perhaps two ounces of fluid, I found five or six hairs, from eight to ten inches long.

Now I do not mention this case as being the first, or the only case of the kind on record, because I know better. But as one that is not very often met with in common practice, and of the importance of making ourselves acquainted with all cases as far as possible, although the patient may object to the means made use of, as life may be saved by a certain course of independence on the part of the Physician.

